# Gut microbiota and its therapeutic implications in tumor microenvironment interactions

**DOI:** 10.3389/fmicb.2024.1287077

**Published:** 2024-01-23

**Authors:** Pengya Feng, Xia Xue, Ihtisham Bukhari, Chunjing Qiu, Yingying Li, Pengyuan Zheng, Yang Mi

**Affiliations:** ^1^Key Laboratory of Helicobacter Pylori, Microbiota and Gastrointestinal Cancer of Henan Province, Marshall Medical Research Center, Fifth Affiliated Hospital of Zhengzhou University, Zhengzhou, China; ^2^Department of Children Rehabilitation Medicine, Fifth Affiliated Hospital of Zhengzhou University, Zhengzhou, China; ^3^Department of Gastroenterology, Fifth Affiliated Hospital of Zhengzhou University, Zhengzhou, China

**Keywords:** cancer therapy, gut microbiota, microbiota tumor microenvironment, synthetic biology, therapeutic target

## Abstract

The development of cancer is not just the growth and proliferation of a single transformed cell, but its tumor microenvironment (TME) also coevolves with it, which is primarily involved in tumor initiation, development, metastasis, and therapeutic responses. Recent years, TME has been emerged as a potential target for cancer diagnosis and treatment. However, the clinical efficacy of treatments targeting the TME, especially its specific components, remains insufficient. In parallel, the gut microbiome is an essential TME component that is crucial in cancer immunotherapy. Thus, assessing and constructing frameworks between the gut microbiota and the TME can significantly enhance the exploration of effective treatment strategies for various tumors. In this review the role of the gut microbiota in human cancers, including its function and relationship with various tumors was summarized. In addition, the interaction between the gut microbiota and the TME as well as its potential applications in cancer therapeutics was described. Furthermore, it was summarized that fecal microbiota transplantation, dietary adjustments, and synthetic biology to introduce gut microbiota-based medical technologies for cancer treatment. This review provides a comprehensive summary for uncovering the mechanism underlying the effects of the gut microbiota on the TME and lays a foundation for the development of personalized medicine in further studies.

## 1 Introduction

Cancer is one of the significant causes of death, affecting millions of people globally (Siegel et al., 2019). Only 5–10% of cancer cases are associated with genetics, while most are related to environmental factors (Anand et al., 2008). The tumor microenvironment (TME) has been confirmed to play an essential role in tumor initiation and development, with its interactions with cancer cells well-studied (Kise et al., 2016). Most scientists believe that the TME can offer efficient and cost-effective therapeutics for various cancers, including gastric and colon cancers (Merlo et al., 2006). The TME comprises noncellular components and noncancerous host cells, including endothelial cells, fibroblasts, immune cells, and even microbes (Whisner and Athena Aktipis, 2019). The chemopathological qualities of the TME were classified into six categories, contributing to an in-depth understanding of its complexity and heterogeneity and guiding anticancer therapy (Jin and Jin, 2020). Considering the strengthened comprehension of the essential effects of TME on tumor growth and therapeutic resistance, therapeutic benefits in cancer patients have been achieved by targeting components of the TME (Xiao and Yu, 2021). Therefore, a comprehensive understanding of the TME provides a framework for preventing and treating cancers.

Microbiota is one of the cellular components in TME that play an essential and irreplaceable role in human systems as well as other factors like genetic due to the microbiota community can modulate various biological processes including cellular metabolism, physiology, and immune responses (Marsland et al., 2015; Andreeva et al., 2020). Disturbances in the human microbiota have been linked to several diseases, such as inflammatory bowel diseases (IBDs), cardiovascular diseases, and cancer (De Martel et al., 2012). Microbiota can form the TME for tumor initiation and development by regulating mucosal immunity and hormonal elements in humans (De Martel et al., 2012). Modulating host-microbiota interactions, especially in the gut, which hosts the most rich and diverse microbiota, has emerged as a state-of-the-art therapeutic approach for cancer treatment (Schwabe and Jobin, 2013). Previous studies have shown that the gut microbiota can regulate the sensitivity and responses of cancer patients to chemotherapy (Liu et al., 2022; Rahman et al., 2022). Furthermore, alterations in gut microbial structure have been reported to serve as potential indicators for early cancer diagnosis and other diseases (Sepich-Poore et al., 2021). Therefore, a comprehensive understanding of the interaction between the gut microbiota and the TME is beneficial for developing effective, safe, and patient-friendly treatments.

In this review, we (1) investigated the effects of microbiota and the TME on host immunity; (2) introduced their mutual effects on cancer prevention and therapy; and (3) discussed various methods for adjusting the TME to maximize the therapeutic effect of cancer, including fecal microbiota transplantation (FMT), dietary adjustments, and synthetic biology design. This study will provide a foundation for cancer-targeted therapies based on the gut microbiota and the TME in future applications and studies.

## 2 Role of the gut microbiota in cancer

### 2.1 Human microbiota

The human body hosts various microbes, with over 100 trillion symbiotic microorganisms (Sender et al., 2016). The human microbiota comprises complicated communities of bacteria, archaea, and viruses (Matson et al., 2021). The primary colonizers in these communities belong to six phyla: Firmicutes, Bacteroidetes, Proteobacteria, Actinobacteria, Fusobacteria, and Cyanobacteria (Ghosh and Pramanik, 2021). However, the relative abundance and load of these phyla, especially the bacterial composition at the genera level, differ significantly among the different communities (Cho and Blaser, 2012). Each anatomical niche, including the skin, gut, vagina, nose, mouth, and conjunctiva, possesses a distinct mixture of microbial populations. Among these, the human microbiota, especially the gut microbiota, has gained more attention due to its significant effect on human health and diseases (O'Hara and Shanahan, 2006). However, the relationship between microbiota and tumorigenesis is complicated as it is influenced by the microbial community and abiotic factors. Studies have reported that changes in the gut microbial community and its homeostasis can influence the development and progression of multiple cancers in humans. Chronic inflammation caused by the gut microbiota is a widely accepted mechanism that promotes tumor development. Furthermore, substances released by gut bacteria have been found to damage DNA, resulting in pathogenic mutations (Kumar et al., 2023). Notably, certain species of gut bacteria exhibited antitumor effects in some animal studies, particularly those involved in short-chain fatty acid (SCFA) synthesis (Yao et al., 2022). Moreover, gut bacteria can enhance the immune response to tumors by activating the immune system (Ge et al., 2021). By studying the tumor-associated gut microbiota, cancer prognosis can be predicted, and thus, stopping the generation of these associated microbes can halt cancer progression. Research on this microbiota would provide a novel and more patient-friendly strategy for cancer treatment.

### 2.2 Cancer microbiota

The International Association for Cancer Research has identified 11 microbes as human carcinogens or “oncomicrobes,” including Human Papillomaviruses, Hepatitis B virus, Hepatitis C virus, Epstein–Barr virus, Human T-cell lymphotropic virus type I, Human immunodeficiency virus type 1, Human herpesvirus 8, Merkel cell polyomavirus, *Helicobacter pylori* (*H. pylori*), *Opisthorchis viverrini*, and *Schistosoma haematobium* (Plummer et al., 2016). Among these, *H. pylori*, regarding gut microbiota modulation, has received significant attention and has been well studied. *H. pylori* is known to cause chronic inflammation of the gastric mucosa, potentially leading to gastric and duodenal ulcers, and is confirmed to be related to mucosa-associated lymphoid tissue lymphoma (extranodal marginal zone B-cell lymphoma) in the stomach (Wang et al., 2014). Consequently, since gastric cancer caused by *H. pylori* infection depends significantly on the long-term inflammatory response of the host immune system, understanding the relationship between *H. pylori* and other gastric bacterial infections and host immune responses at the molecular level during gastric carcinogenesis is of great importance (Kim and Wang, 2021).

The molecular mechanisms underlying the epidemiology of oncomicrobes and their clinical scenarios have been well studied (Sepich-Poore et al., 2021). Although carcinogenic microbiota can colonize various parts of the human body, their detection in microbial-triggered cancer is challenging, mainly due to individual differences in genetic makeup (Ribet and Cossart, 2015). In addition, certain microbiota can cause cancer through genotoxin-mediated mutagenesis, such as colibactin (Wilson et al., 2019) and cytolethal distending toxins, indicating that not all microbiota are carcinogenic or can be conditionally carcinogenic, e.g., prolonged *H. pylori* infection can trigger gastric cancer (Parsonnet, 1993; Matysiak-Budnik and Mégraud, 2006).

Increasing evidence shows that a significant “complicit” microbiota can trigger carcinogenesis through interactions with other abiotic factors. This category encompasses multiple immunomodulatory roles of microbiota and their bioactive metabolites involved in tumor growth, which might be related to the effect of the immune system on solid tumorigenesis (Bagheri et al., 2022). Tumors located on boundary surfaces-including the oropharynx, skin, and the respiratory, digestive, and genitourinary tracts-contain microbiota, which complicates cancer-microbe causality (Garrett, 2015). The gut microbiota establishes the core factors of the gut microenvironment under healthy and cancerous conditions. Simultaneously, different TMEs show diverse community structures of the central gut microbiota. The different gut microbiota compositions associated with various cancers are summarized in Table 1. Moreover, a decrease in the abundance of specific microorganisms may also increase the cancer risk of the host in areas far from the transfer of such microorganisms (Sears and Garrett, 2014). Therefore, understanding microbes throughout the body is essential for understanding the relationship between the gut microbiota and cancer.

**Table 1:** Gut microbiota compositions are associated with cancers.

Type	Experimental Model	Bacterial species	Virulence factor	Mechanisms	Reference
Colorectal cancer	Mice	*Peptostreptococcus anaerobius* (Firmicutes)	N/A	Promotion of cell proliferation, induction of oxidative damage, TLR2/TLR4 interaction, SREBP2/AMPK activation	Tsoi et al. (2017)
	Mice	*Fusobacterium nucleatum* (Fusobacteria)	FadA	Cell proliferatio induction, modulation of Ecadherin/β-catenin signals, enhanced expression of NF-κB, cyclin D1	Rubinstein et al. (2013)
	Mice	Genotoxic *Escherichia coli*	Colibactin	Phosphorylated H2AX foci formation	Cuevas-Ramos et al. (2010)
	Stool sample	*Enterotoxigenic Bacteroides fragilis* (Bacteroides)	*B. fragilis* toxin, fragilysin	Elevated IL-1 levels, activation of STAT3/β-catenin, Ecadherin cleavage, induction of Th-17 response	Ulger Toprak et al. (2006)
Pancreatic cancer	Mouse and macrophages	*Porphyromonas gingivalis* (Bacteroidetes)	N/A	Apoptosis induction, interaction with TLR2/TLR4, activation of STAT3/NF-κB signaling pathways	Huck et al. (2017)
Liver cancer	Mouse	*Helicobacter hepaticus* (Proteobacteria)	Cytolethaldistending toxin	Promotion of endoreplication, enhanced p-21/Ki-67 expression	Péré-Védrenne et al. (2016)
Gastric cancer	Mouse and epithelial cells	*Helicobacter pylori* (Proteobacteria)	VacA	Autophagy induction, elevated MAPK/ERK1/2 expression, activation of Wnt/β-catenin signals	Meng et al. (2018)

Given the high individual heterogeneity of the gut microbiota due to variations in genetics, diet, and other factors, its performance varies across subtypes of certain cancers. The gut microbiota is highly related to chronic inflammation in multiple organs, which can promote the development and progression of tumorigenesis. However, it is unrelated to cancers resulting from genetic inheritance or mutations (Karki and Kanneganti, 2019). Previous research has found that *Enterobacteriaceae* exhibits high abundance across all subtypes of gastric tumors, whereas *Lachnoclostridium*, *Bifidobacterium*, *Parabacteroides*, and *Barnesiella* are found in patients with adenocarcinoma (Zhou et al., 2021). The gut microbial community and biodiversity significantly depend on the types/subtypes of tumors and different tumor stages (Chen et al., 2022). Although the role of the gut microbiota in subtypes of different cancers requires further study and clarification, its potential for tumor diagnosis and treatment has gained widespread recognition.

## 3 Relationship between the gut microbiota and the TME

### 3.1 The microbiome as an ingredient of the tumor microenvironment

**Figure 1:**
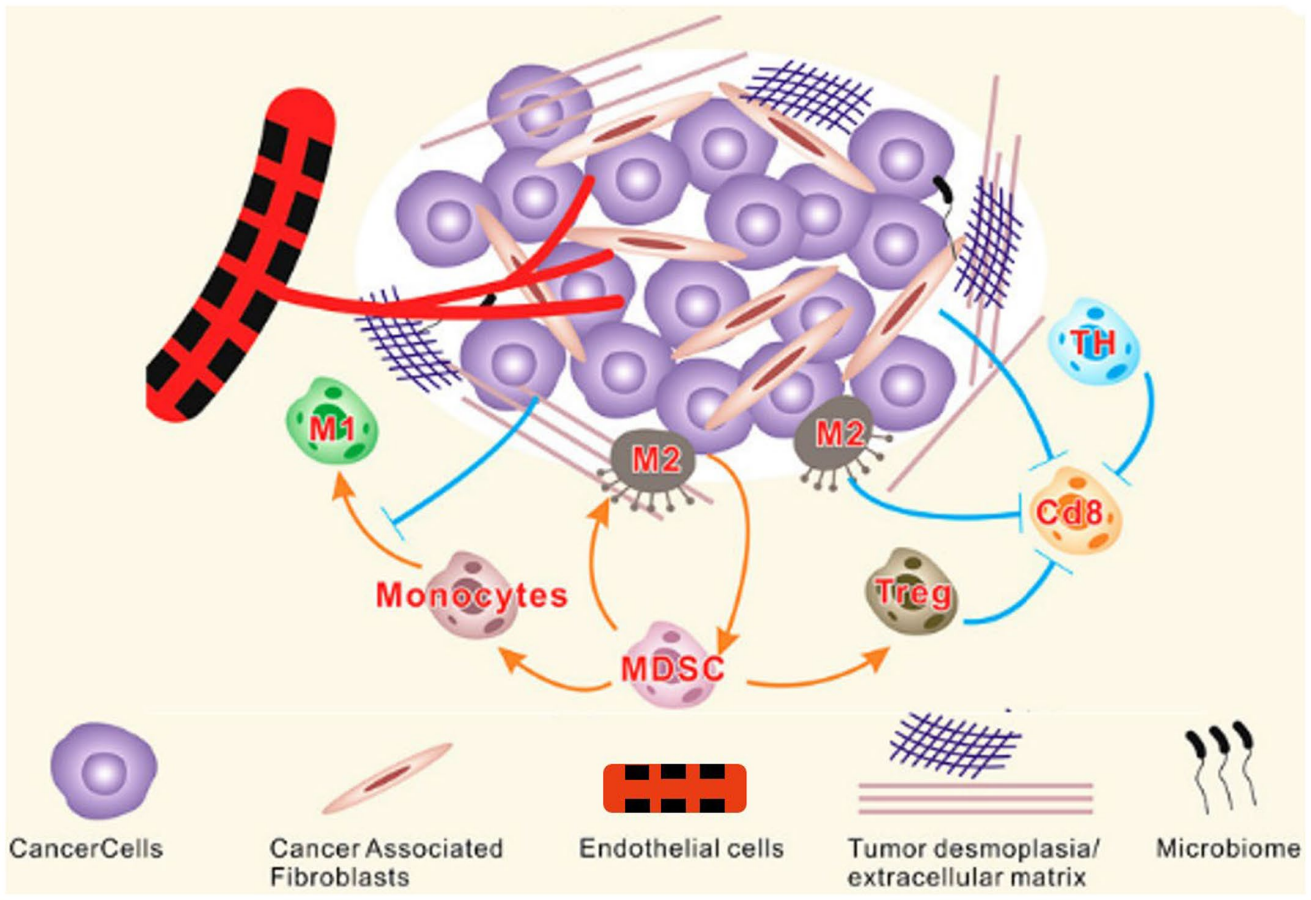
Components of TME. The TME is a complex network of stromal cells, microbiome and other cellular entities surrounding tumor cells. Tumor and stromal cells actively interact to support tumor growth by promoting desmoplasia, angiogenesis, and immune suppression. MDSC, Myeloid-derived suppressor cells; TH, T helper cells; M, Macrophages (Zubair et al., 2022).

The TME comprises malignant and nonmalignant cells and the contents of the tumor (Figure 1). The permanent mutual relationship between tumor cells and the TME significantly affects tumor initiation, progression, metastasis, and therapeutic responses (Xiao and Yu, 2021). Recently, the conventional drugs including aspirin, celecoxib, β-adrenergic antagonist, metformin, and statin with antitumor capability that show potential use in combination therapy by targeting TME components (Jin and Jin, 2020). Due to the different layers of microbial niches, the TME is a complex environment in which the microbiota has been recognized as a novel yet essential element (Turroni et al., 2008; Rowland et al., 2018). The microbiota functionally reduces tumor cell metabolism, such as inflammation, genotoxin generation, and production of bacterial metabolites with various characteristics (Kovács et al., 2020). Accumulating evidence has shown that the interactions between the microbiota and their metabolites in the TME can influence host immunity and the intestinal epithelium, ultimately driving or inhibiting tumor growth (Barry et al., 2018).

The model of the gut microbiota and the TME is complex, including biotic and abiotic drivers from cells, blood vessels, and the extracellular matrix that constitutes the tissues surrounding a tumor (Zhu et al., 2021). Studies have reported that gut bacteria can regulate the activation of human immune cells to migrate to the TME for tumor cell elimination (Buzas, 2023). In addition, the complex interaction between the gut microbiota and the TME can enable tumor cells to evade the immune system and proliferate more efficiently (Kalaora et al., 2022). Understanding this system holds promise for cancer prevention, diagnosis, and treatment.

### 3.2 Effects of the gut microbiota on the TME

The gut microbiota is crucial in the development, maintenance, and growth of the host immune system (Shi et al., 2017). The intestinal ecosystem can affect local and distant neoplasia by influencing the influx of myeloid, immune context, lymphoid cells, and inflammatory and metabolic patterns (Ma et al., 2019). Thus, the gut microbiota is emerging as a critical modulator of the TME in various cancers, such as colorectal, gastric, and liver cancers (Lakritz et al., 2014). For instance, a previous study reported that bacteria such as *Fusobacterium nucleatum* can enhance tumor growth by inhibiting human immune responses (Chattopadhyay et al., 2021). Moreover, breast and ovarian cancers are associated with specific biosignatures of the gut microbiota, such as the abundance of *Lactobacillus crispatus*, which negatively correlates with cancer occurrence (Banerjee et al., 2018).

**Figure 2:**
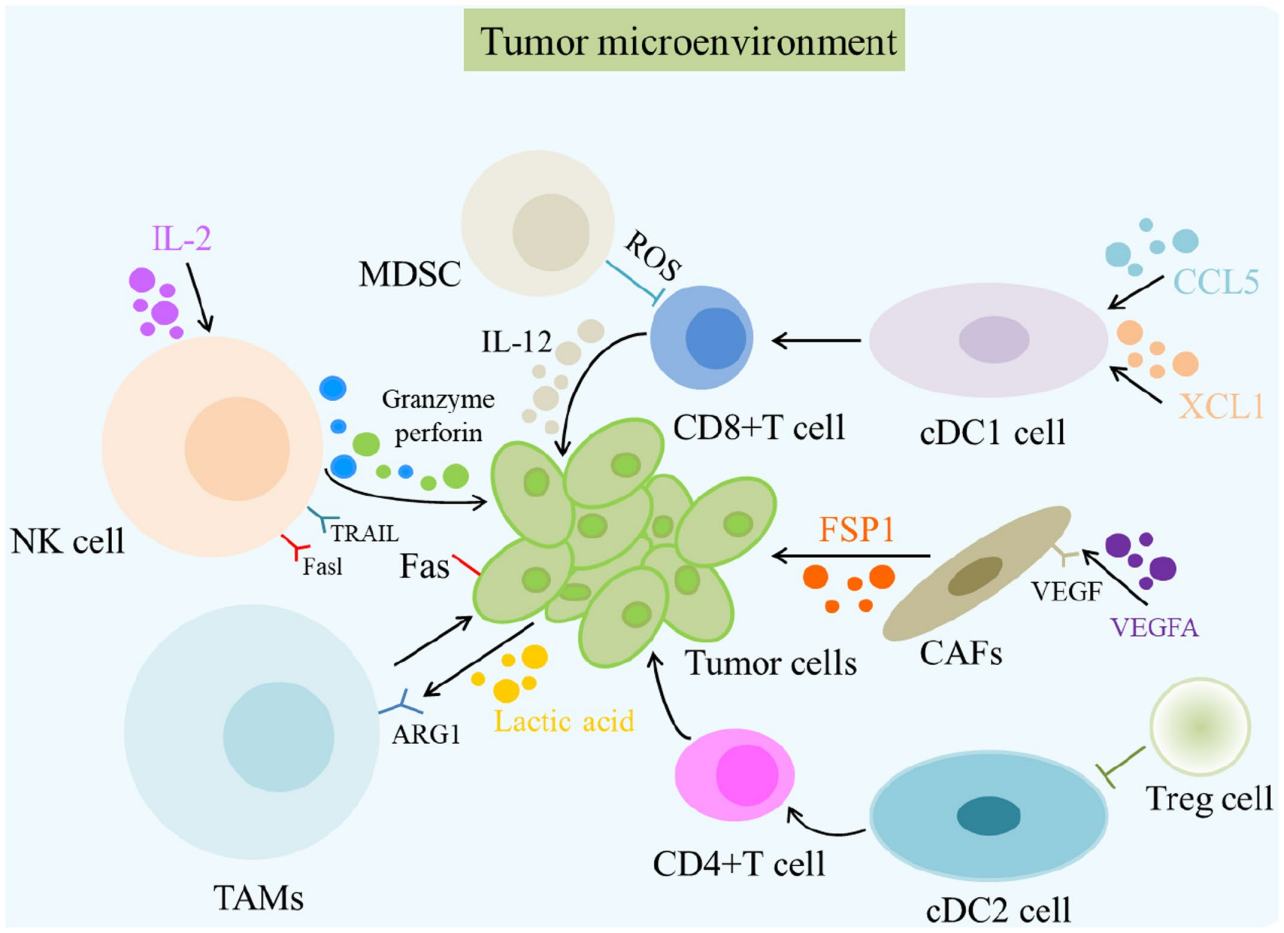
The role of TME in cancer and its immunotherapy. The primary cells of the TME in cancer immunity are NK cells, DC cells, CD8 + T cells, Treg cells, fibroblasts, TAMs, and MDSCs. Different cells induce the death of tumor cells in various ways, such as releasing perforin and granzyme and mediating cytotoxicity by TRAIL and Fasl receptors. MDSC, Myeloid-derived suppressor cells; ROS, Reactive oxygen species; NK cells, Natural killer cells.

Furthermore, the secretory components of the gut microbiota are reported to be associated with TME. For example, outer membrane vesicles (OMVs) can reprogram the TME toward a pro-TH1 pattern (CXCL10, IFN-g; Kim et al., 2017). Metabolites produced by the gut microbiota, including butyrate and niacin, can mediate Gpr109a-dependent interleukin (IL)-18 induction in the colonic epithelium, suppressing colitis and colon cancer. Additionally, the TME can regulate tumor development, metastatic progression, and the efficacy of therapeutic interventions (Figure 2; Singh et al., 2014). Studies have found that tumor cells can establish a bidirectional functional relationship with the surrounding stromal cells during malignant progression (Poutahidis et al., 2013). The synthesis and secretion of sonic hedgehog, which selectively reacts with stellate cells, are promoted by the activation of the CXCL12/CXCR4 pathway in pancreatic tumor cells, thereby driving desmoplasia (Singh et al., 2012). The desmoplastic TME affects pancreatic cancer pathobiology and chemoresistance (Özdemir et al., 2014). One study on lung cancer showed that cancer-associated fibroblast (CAF)-derived IL-6 induces epithelial-mesenchymal transition and confers resistance to cisplatin in non-small cell lung carcinoma (Yang et al., 2016). Moreover, CAFs secrete various proinflammatory molecules (IL-6, CCL2, and TGF-β), which enhance immunosuppressive cell recruitment (Dirkx et al., 2006).

High accumulation of tumor-associated macrophages (TAMs) and other immunosuppressive cells in the TME induces cancer progression and therapy resistance (Xiang et al., 2021; Yan and Wan, 2021). The depletion of CD163+ TAMs, which cause immune suppression, leads to robust infiltration of cytotoxic T cells into the TME, resulting in the control of melanoma development (Etzerodt et al., 2019). High CD163^+^ TAMs in the TME have been linked to worse clinical outcomes in patients with various myelomas (Omatsu et al., 2014). Pancreatic tumors exhibit a growing infiltration of TAMs and a scarcity of cytotoxic T cells in their TME (Lankadasari et al., 2019). Additionally, the effects of TAMs on angiogenesis have been previously reported (Larionova et al., 2021). For example, TAM depletion can induce a significant decrease in vessel density (Yang et al., 2016). Thus, various factors, such as MMPs, ILs, VEGF, PDGF, and TGF-β secreted by TAMs in the TME, can promote vascularization in tumor tissues (Dirkx et al., 2006). MDSCs are used to heavily infiltrate the TME of glioblastoma, activating B-cell–induced immune suppression by inhibiting CD8^+^ T-cell activation (Lee-Chang et al., 2019). Furthermore, the high intratumoral burden of *F. nucleatum* correlates with a poor response to neoadjuvant chemotherapy in patients with esophageal squamous cell carcinoma (Yamamura et al., 2019). Therefore, the TME microbiota significantly influences cancer pathogenesis and therapeutic outcomes.

Noncellular components of the TME are also crucial for cancer progression, aggressiveness, and chemoresistance (Schulz et al., 2019). The stiffness of the extracellular matrix promotes tumor cell survival and proliferation while upregulating integrin signaling (Pickup et al., 2014). Hyaluronic acid, a CD44 receptor, is abundant in the TME of various cancers (Mattheolabakis et al., 2015). Their mutual effects activate cancer-promoting signaling pathways and induce the upregulation of noncoding RNA species, such as miR-10b/miR-302/miR-21 and lncRNAs. In pancreatic cancer, the stroma is highly reactive with different hyaluronic acids, resulting in elevated interstitial fluid pressures that lead to vascular collapse and poor chemotherapy outcomes (Provenzano et al., 2012). Targeting enzymes in pancreatic tumors with recombinant hyaluronidase has been shown to degrade hyaluronic acid and enhance therapy by reducing metastasis and improving survival (Kim et al., 2021). In addition, the secretory components of the gut microbiota are related to the TME. For instance, OMVs can reprogram the TME toward a pro-TH1 pattern (CXCL10, IFN-g; Kim et al., 2017), while metabolites, such as butyrate and niacin, can mediate the Gpr109a-dependent induction of IL-18 in the colonic epithelium, suppressing colitis and colon cancer.

Metabolites from the gut microbiota enter host cells and interact with the human immune response, promoting various tumor-inhibitory and immunomodulatory molecules. They also inhibit inflammation by maintaining the integrity of the epithelial barrier and the intestinal tract (Rooks and Garrett, 2016). A previous study found that the products of the metabolic activities of the gut microbiota significantly affect host metabolic pathways related to adiposity, lipids, and energy homeostasis (Poutahidis et al., 2013). Thus, uncovering how metabolites and submetabolites from the gut microbiota affect immune cells and reshape the TME can strongly contribute to the development of tumor therapeutics.

Gut microbiota metabolites, such as SCFAs and inosine, directly or indirectly interact with the TME to reshape it, thereby affecting the cancer process (Min et al., 2005). SCFAs contribute to maintaining intestinal homeostasis and regulating intestinal barrier function (Wang et al., 2017). Moreover, several fatty and cholic acids are associated with inflammation (Min et al., 2005). Butyrate and SCFAs, which can be generated by *Faecalibacterium prausnitzii*, control angiogenesis and reduce the expression of proangiogenic factors. Thus, increasing butyrate concentration is believed to slow down and halt cancer growth (Davie, 2003). Conversely, deoxycholic and petrocholic acids can cause DNA damage by increasing the generation of reactive oxygen species (Payne et al., 2007). Recent studies have shown that the intestinal bacteria *B. pseudolongum* can produce inosine, which drives Th1 cell differentiation in the presence of exogenous IFN-g (Kroemer and Zitvogel, 2020). Moreover, the status of *B. pseudolongum* has been reported to be associated with the response to ICB therapy, such as anti-CTLA-4 and anti-PD-L1, through its interaction with the adenosine A2A receptor on T cells (Mager and Burkhard, 2020). CTLA-4 and PD-L1 are the primary targets of immune checkpoint therapy, which involves membrane-bound molecules that impede unbounded T-cell responses after initial stimulation (Mager et al., 2020). Thus, cancer cells can avoid immune surveillance by employing this mechanism. However, while reactivating inefficient T cells, immune checkpoint inhibitors (ICIs) can restore the response to tumor antigens (Sharma and Allison, 2015). Clinical research and preclinical trials have revealed that the gut microbiota affects the efficacy of ICIs, thereby explaining significant variations in patients’ responses to ICIs (Vétizou et al., 2015). Hence, gaining an in-depth understanding of how the gut microbiota, their metabolites, and the host immune system interact to reshape and regulate the TME holds promise for advancing cancer immunotherapy.

Overall, the effects of the gut microbiota on the TME are complex and not yet fully understood. However, studies on this system have demonstrated its potential application in manipulating gut microbes to influence the effectiveness of cancer treatment and improve patient outcomes.

## 4 Gut microbiota modulation and their TME target

### 4.1 Cancer diagnostics based on microbiota

Cancer is typically diagnosed following the identification of a lump through palpation or imaging techniques, followed by a biopsy to confirm cellular malignancy (Fass, 2008). Tomographic detection techniques, including PET, MRI, and CT, efficiently identify macroscopic lesions in the body (Pokharel et al., 2013). Compared to stable genetic characteristics, the homeostasis of the human gut microbiota is more susceptible to tumorigenesis. Furthermore, studies have confirmed that the gut microbiota dynamics can potentially aid in diagnosing and locating malignancies, such as *Streptococcus gallolyticus* bacteremia, based on their gastrointestinal origin (Klein et al., 1977). Most microbial-based cancer diagnostics focus on sequencing tumors within the aerodigestive tract, including colorectal (Flemer et al., 2017), pancreatic (Farrell et al., 2012), and lung cancer (Yan et al., 2015). It has been suggested that different cancer types may host microbiota with unique compositions outside the aerodigestive tract, such as in the oral cavity. Nejman et al. investigated intratumoral microbiota from over 30 cancers, applying their blood-based diagnostics and providing visual evidence of microbial intratumoral spatial distributions and intracellular localization in seven different cancers (Nejman and Livyatan, 2020).

Currently, several bacteria-based strategies have been developed for tumor detection (Panteli et al., 2015), including the use of engineered bacteria that combine the specificity of tumor-targeting bacteria with the sensitivity of biomarker assays (Panteli et al., 2020). Attenuated bacteria were engineered to release an exogenous reporter protein, ZsGreen, using a remotely inducible genetic switch (Kaimala et al., 2018). Both *in vivo* and *in vitro* experiments showed that these bacteria could identify tumors through systemic measurements of the released ZsGreen (Panteli et al., 2020). Although bacteria-based cancer diagnosis is a promising strategy, it faces several challenges, such as low biomass relative to the host and confounding from reagents or environmental pollutants. Thus, combining gut microbiota-based methods with conventional diagnostic techniques, including genome sequencing, qPCR, immunohistochemistry, and electron microscopy, can offer more accurate and efficient cancer diagnoses.

Microbial-based cancer diagnostics have emerged as a new area that is focused on designing or developing novel strategies based on specific biosignatures of the gut microbiota in various cancers or at different tumor stages (Kim and Lee, 2021). Furthermore, deep learning and machine learning algorithms enable the identification of microbial profiles indicative of cancers, which is the basis of precision medicine. Microbial-based cancer diagnostics also hold the potential to improve cancer screening and early detection efforts, promising the development of more accurate and effective diagnostic tools for various cancers and ultimately improving patient outcomes.

### 4.2 Microbial-based cancer therapy

**Figure 3:**
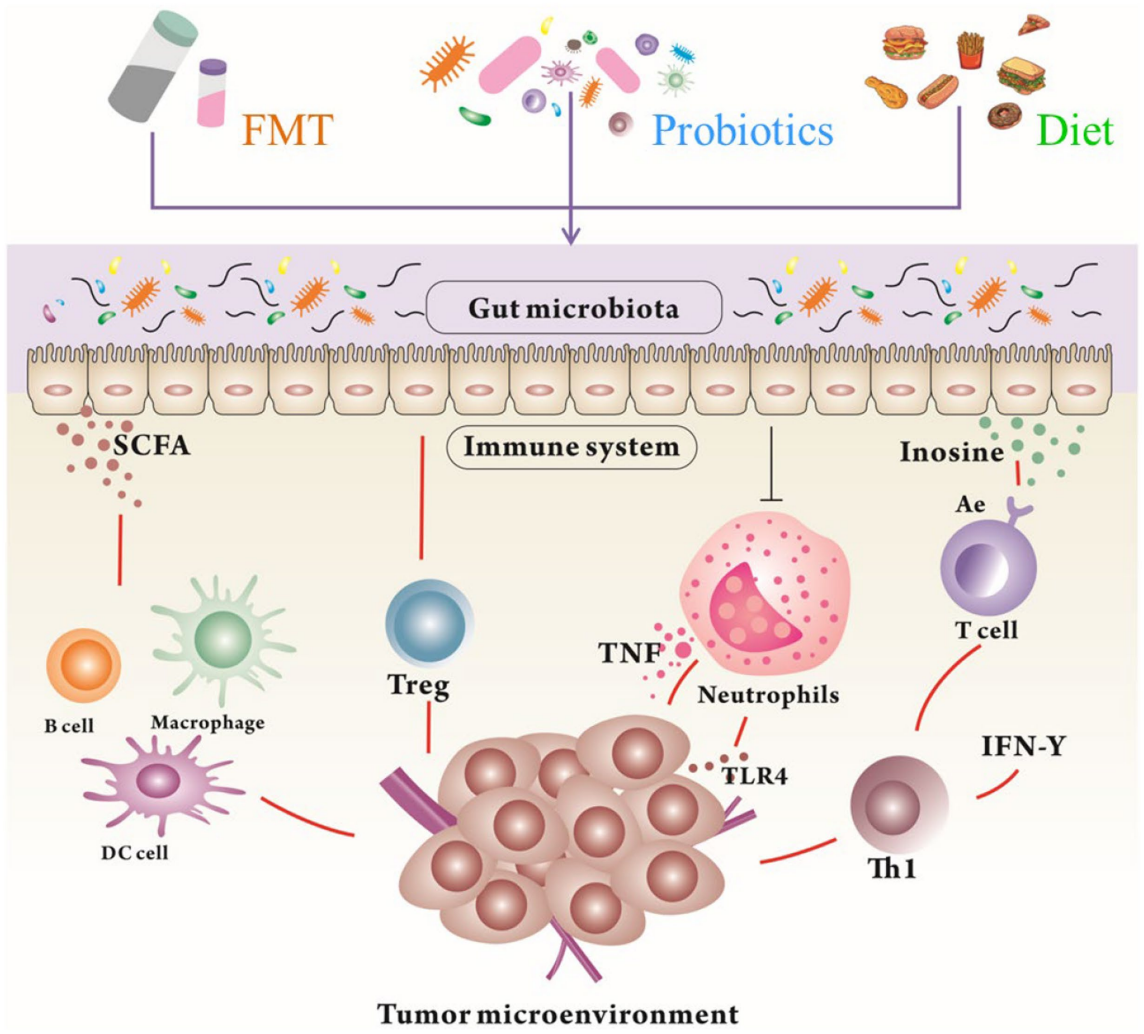
The modulation of the gut microbiota and their metabolites by FMT, probiotics, and diet to reshape TME for tumor therapy. Gut microbiota and their metabolites can promote the immunotherapy in humans through different mechanisms. FMT, Fecal Microbial Transplantation; SCFA, Short Chain Fatty Acids; TNF, Tumor necrosis factor.

The human gut microbiota is recognized as a fundamental component of the immune system (Hooper and Macpherson, 2010; Maynard et al., 2012). Further studies have demonstrated that the gut microbiota can regulate immune responses, thus affecting the efficacy of cancer immunotherapy (Roy and Trinchieri, 2017). Several clinical trials have recently been conducted to alter the gut microbiota for cancer therapy (Table 2). Methods such as FMT, probiotics, dietary interventions (discussed in the subsequent section), and microbial engineering based on synthetic biology offer potential anticancer effects by targeting both tumor cells and the TME (Figure 3).

**Table 2:** Selected clinical trials are modulating the gut microbiota in cancer therapy.

Type	Patient number	Objective	Intervention	Clinical outcomes	NCT number
Melanoma	20	To study concurrent use of FMT and pembrolizumab in patients with PD-1-resistant melanoma	FMT (donor responder to PD-1 therapy) with pembrolizumab	Overall response rate, change in T cell composition and function; change in innate and adaptive immune subsets	NCT03341143
Breast cancer	20	To assess the efficacy of Presurgical antibiotics to influence antitumor immune function	Primal Defense ULTRA Probiotic Formula	Mean number of cytotoxic CD8^+^ T cells	NCT03358511
Colorectal cancer	35	To investigate the effect of probiotics on gut microbiota and the immune and inflammatory response	Probiotics	The colonic microbiota GI function	NCT00936572
Colon cancer	20	To reactivate the tumor-suppressor genes using probiotics	ProBion Clinica (*Bifidobacterium lactis*, *L. acidophilus*)	Changes in microbiota composition and DNA methylation	NCT03072641
Acute myeloid leukemia	20	To use FMT to prevent complications associated with dysbiosis in patients undergoing intensive treatment	Auto-FMT	Dysbiosis correction, eradication of multidrug resistant bacteria, definition of dysbiosis biosignature	NCT02928523
Hepatocellular carcinoma	64	To assess the role of probiotics in preventing septic and liver functional complications related to bacterial translocation following surgical resection of HCC	Probiotics-Lactibiane Tolerance (*Bifidobacterium lactis*, *L. acidophilus, L. plantarum, L*.*salivarius*)	Area under the plasma concentration versus time curve of endotoxin circulating levels	NCT02021253
Lung cancer	41	To assess the effects of chemotherapy on microbiome and probiotics on chemotoxicity	*Clostridium butyricum*	Composition of microbiome with probiotics, adverse effects of chemo, change in immunity and nutrition index	NCT02771470
Renal cell cancer	20	To prevent diarrhea in patients treated with sunitinib by probiotics	Micronutrient- fortified probiotic yogurt	Change in levels of *Bifidobacterium* spp. in stool samples	NCT02944617

FMT is an artificial strategy for manipulating the gut microbiota and the TME (Zhang et al., 2020). Several clinical conditions, such as *clostridium difficile* infection, ulcerative colitis, and other gastrointestinal conditions, have been successfully treated by transferring fecal material from a donor to a recipient through colonoscopy, enema, or oral administration (Tan and Johnson, 2019). According to ongoing clinical trials, FMT from donors responsive to immunotherapy can enhance antitumor immune responses and potential clinical outcomes (Kang and Cai, 2021). Modifying the gut microbiota through FMT has been found to modulate the composition of the tumor microbiota, antitumor immune responses, and tumor growth kinetics (Matson et al., 2021). However, the long-term effectiveness and stability of FMT remain unclear (McQuade et al., 2019). Although some clinical trials have successfully incorporated the modulation of the gut microbiota using FMT into cancer therapy, the applications of FMT in cancer patients are limited due to antibiotic preconditioning, administration route, and modulation frequency, complicating its clinical use in targeting the gut microbiota (Cheng et al., 2020). Therefore, more specific clinical trials are needed for fecal transplants in cancer patients.

Probiotics are widely used to shift the microbial community (Zaramela et al., 2021). These interventions are being investigated for tumor therapy based on both retrospective studies and prospective clinical trials (Panebianco et al., 2020). Live bacteria are orally administered in probiotics, supplying substrates that stimulate the development or activity of beneficial bacteria in the gut and that further modulate the components of the overall gut microbiota (Markowiak and Śliżewska, 2017). Recent findings have confirmed that the gut microbiota can regulate immune responses, which could potentially affect the efficacy of cancer immunotherapy, indicating that probiotics can reduce the side effects of anticancer therapy (Lu et al., 2021). Several commercially available probiotics have been studied in preclinical models and clinical trials (Helmink et al., 2019). It has been reported that patient outcomes can be influenced by compositional variations in the gut microbiota or the TME (Helmink et al., 2019).

Anaerobic bacteria play a crucial role in the gastrointestinal tract (Zaramela et al., 2021). A functional gut microbiota Bifidobacterium is commonly used to treat IBDs, including ulcerative colitis (Zhang et al., 2021). The TME creates a suitable growth environment for anaerobic bacteria under low-oxygen conditions (Leppäranta et al., 2008). The antitumor effects of anti-CD47 immunotherapy can be significantly improved by accumulating Bifidobacterium in the TME (Sivan et al., 2015). Current clinical trials primarily focus on the effectiveness of probiotic treatment for colorectal, kidney, breast, gynecologic, and lung cancers.

Microbial metabolites also contribute to regulating antitumor immunity (Sipe et al., 2020). SCFAs are crucial for maintaining gut integrity and serve as the primary energy source for intestinal epithelial cells (Parada Venegas et al., 2019). SCFAs, such as acetate, propionate, and butyrate, are absorbed through the intestinal epithelium and transmitted to T cells through G-coupled protein receptors to influence tumor differentiation (Moniri and Farah, 2021). In the colon, SCFAs protect gut integrity from invading foreign microorganisms by inducing Treg cells or IL-10 (Park et al., 2015). A direct interaction exists between SCFAs and CD8+ T cells in the circulation system, enhancing their antitumor effects (Bachem et al., 2019). Overall, SCFA-producing microbiota contribute to the response to ICIs (Huang et al., 2020). In addition, prebiotics and synbiotics (a combination of probiotics and prebiotics) are ideal for cancer prevention (Raman et al., 2013). Prebiotics are defined as fermentable, nondigestible food ingredients that can improve the health of the host (Legesse Bedada et al., 2020).

Dietary fibers resist digestion and absorption in the small intestine but undergo complete or partial fermentation in the large intestine (Buttriss and Stokes, 2008; Mudgil and Barak, 2013). Most fractions of edible plants or their extracts are carbohydrates and are regarded as prebiotics (Hijová et al., 2019). Fermentation of nondigestible compounds is key to proliferation and apoptosis modulation in tumor cells (Cruz-Bravo et al., 2014). Prebiotics protect cells against cancer through fecal bulking, colonic pH change, carcinogen binding to bacteria, xenobiotic-metabolizing enzymes, gene expression modulation in feces and cecum, and immune response modulation (Harris and Ferguson, 1993). Therefore, diet, lifestyle, and gut microbiota composition are related (Shamekhi et al., 2020). Regarding dysbiosis or bacterial imbalance in the intestine due to variations in diet or the environment, tumors can be induced by virulence factors, microbial metabolites, and inflammatory routes (Dos Reis et al., 2017).

### 4.3 Synthetic biology application on cancer diagnosis and therapy

**Figure 4:**
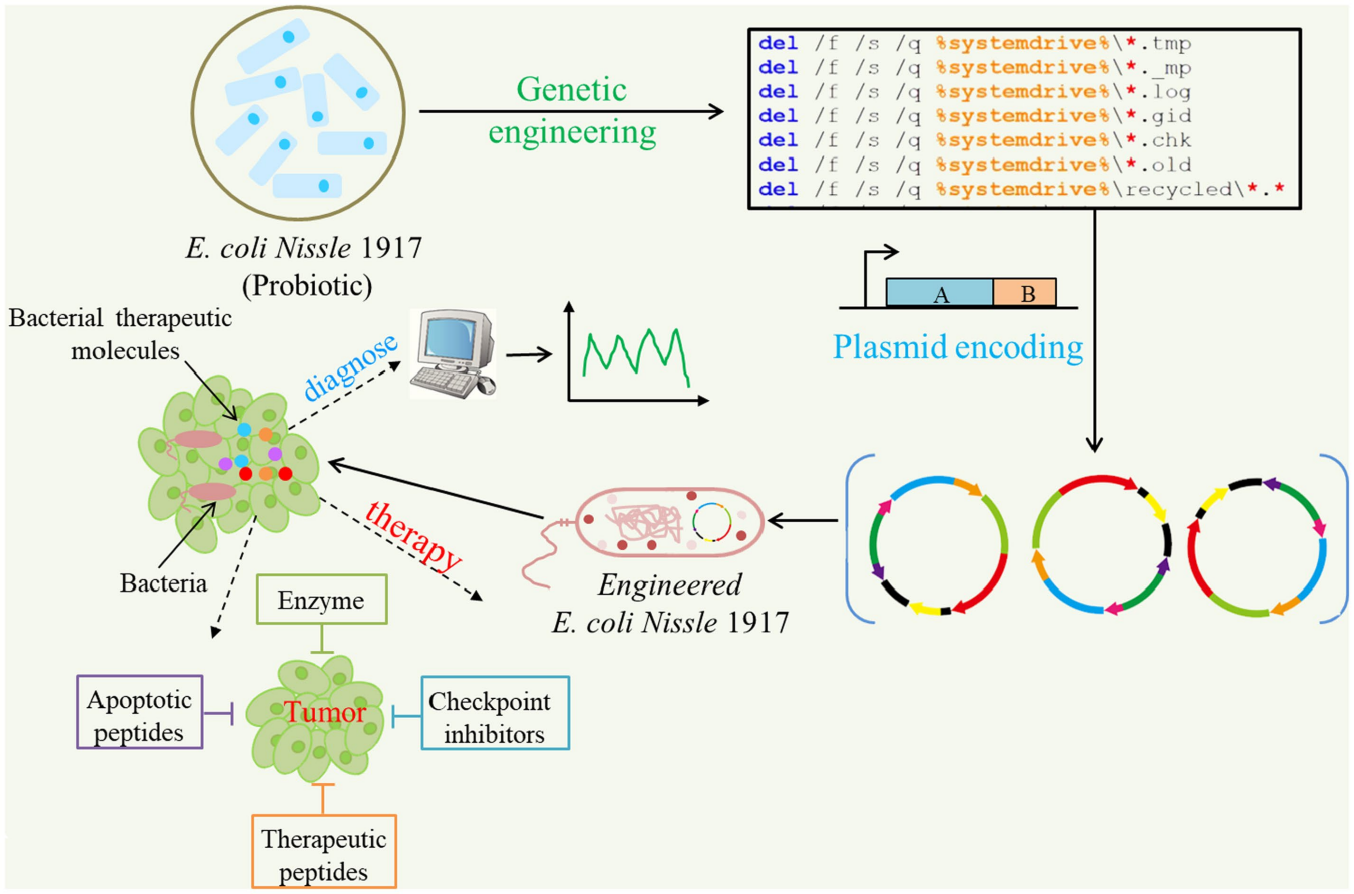
Applications of synthetic biology for cancer diagnosis and targeted therapeutics. Some probiotics like *E. coli Nissle* 1917 can be programmed to produce and deliver anti-cancer agents in solid tumors. Multiple drug payloads can be encoded by one or more engineered strains against tumors.

Synthetic biology has enabled the modification of living cells through sophisticated decision-making processes to achieve user-defined outcomes, such as creating sense-and-respond adaptive therapies (Kitada et al., 2018). Some bacterial species selectively proliferate and accumulate at tumor sites, making them suitable candidates for tumor monitoring and targeted therapy (Figure 4; Kramer et al., 2018). The ability of bacteria to target tumors can be improved through synthetic biology, and therapeutic payloads can be delivered with increased precision. For instance, to decrease off-target effects in healthy tissues, bacteria have been engineered with quorum sensing switches that activate effector gene expression only when the bacterial population reaches a certain threshold density (Anderson et al., 2006). Alternatively, bacteria expressing therapeutic payloads can infiltrate tumor cells or utilize the type III secretory system (T3SS). This syringe-like, protein-based structure injects proteins into target cells (Huh et al., 2013). T3SS has been used to engineer *Salmonella* to deliver antiangiogenic proteins to tumor cells for controlling tumor growth *in vivo* or tumor-related antigens to antigen-presenting cells for triggering antitumor immunity (Shi et al., 2016). In addition, bacteria were programmed to degrade adenosine and kynurenine (West et al., 2018), which inhibit antitumor immunity (Siska and Rathmell, 2015) and produce cyclic-di-AMP (Leventhal et al., 2018), thus activating the stimulator of interferon genes pathway to enhance antitumor immunity (Chen et al., 2016).

Several studies have shown a significant decrease in tumor growth using anticancer-related bacteria in preclinical mouse models (Din et al., 2016). However, bacterial susceptibility was not eliminated by the host’s immune system (Grushkin, 2012). Thus, the use of anticancer-related bacteria may involve sophisticated engineering to ensure their efficient action before they are released by the host’s immune system.

## 5 Prospective and conclusion

The human gut microbiota plays a critical role in tumor growth, progression, and treatment. The interaction among the gut microbiota, host’s immune system, and tumors can offer valuable insights into adjusting the gut microbiota to optimize the TME and enhance cancer immunotherapy.

The human microbiota significantly impacts the overall health of the human host and contributes to the development of various diseases. However, our current understanding of how human microbiota can confer susceptibility to certain cancers remains incomplete. A significant knowledge gap still exists regarding the underlying mechanisms governing bacterial activity as well as the compositions of microbiota due to limitations in culturing many bacterial species, small clinical sample sizes, and a lack of risk assessment data.

Individual diversity in the gut microbiota is the primary challenge for large-scale validation and further intestinal microecology analysis. Therefore, integrating biological information, extensive data, and artificial intelligence into precision medicine would be aid in the future 10 development of novel drugs. However, these methods are not yet widely employed, and further in-depth investigations are necessary to ensure their adoption. In future, personalized medicine will likely incorporate microbiome-based diagnosis and treatment strategies. Despite the current challenges, a powerful new toolkit has been developed by enhancing our understanding of the roles of microbiota in cancer to improve patient care.

## Author contributions

PF: Conceptualization, Writing – original draft. XX: Writing – review & editing. IB: Writing – review & editing. CQ: Data curation, Software, Writing – review & editing. YL: Data curation, Supervision, Writing – review & editing. PZ: Conceptualization, Writing – review & editing. YM: Writing – review & editing.
